# Rare Presentation of Rosai-Dorfman Disease in Soft Tissue: Diagnostic Findings and Surgical Treatment

**DOI:** 10.1155/2022/8440836

**Published:** 2022-03-30

**Authors:** Niteesha Betini, Alana M. Munger, Douglas Rottmann, Andrew Haims, José Costa, Dieter M. Lindskog

**Affiliations:** ^1^Drexel University College of Medicine, Philadelphia, PA, USA; ^2^Department of Orthopaedics and Rehabilitation, Yale School of Medicine, New Haven, CT, USA; ^3^Department of Pathology, University of Michigan, Ann Arbor, MI, USA; ^4^Department of Radiology and Biomedical Imaging, Yale School of Medicine, New Haven, CT, USA; ^5^Department of Pathology, Yale School of Medicine, New Haven, CT, USA; ^6^Department of Pathology, University of Lausanne, Lausanne, Switzerland

## Abstract

*Introduction and Importance*. Rosai-Dorfman disease (RDD) is a rare, benign type II histiocytosis characterized by the infiltration of S100+ histiocytes and emperipolesis. The disease may present in the lymph nodes (nodal RDD), in extranodal sites, or in both nodal and extranodal sites. Among those patients who present exclusively in extranodal sites, only a minority of cases present in the soft tissue. *Case Presentation*. An 18-year-old female presented to orthopedic oncology clinic with a chief complaint of a mass located in her lower back. The patient underwent excision of the lumbosacral mass. Pathologic review demonstrated emperipolesis of lymphocytes and plasma cells within enlarged, eosinophilic histiocytes in a background of lymphoplasmacytic infiltration and collagenous stroma. Immunohistochemical staining demonstrated S100+ and CD163+ histiocytes, consistent with diagnosis of soft tissue RDD. *Clinical Discussion*. Histologically, RDD is generally characterized by emperipolesis—the presence of intact lymphocytes within the histiocyte cytoplasm—and a mixed infiltrate of S100+ histiocytes, mononuclear cells, plasma cells, and lymphocytes. Although soft tissue RDD may histologically resemble nodal RDD, soft tissue RDD also demonstrates some notable histologic differences including the lack of nodal architecture, the presence of increased fibrosis and collagen deposition, and generally fewer RDD cells. *Conclusion*. This case presentation demonstrates one few reports of isolated soft tissue RDD within the lumbosacral region without associated lymphadenopathy or skin changes and highlights the heterogeneity that still exists in the treatment paradigm of extranodal RDD.

## 1. Introduction

Rosai-Dorfman disease (RDD), also known as sinus histiocytosis with massive lymphadenopathy (SHML), is a rare, benign type II histiocytosis characterized by the infiltration of S100+ histiocytes and emperipolesis [1–3]. The disease may present in the lymph nodes (nodal RDD), in extranodal sites, or in both nodal and extranodal sites. Nodal RDD presents with bilateral painless cervical lymphadenopathy and possible involvement of axillary, inguinal, and mediastinal lymph nodes [1–5].

While RDD most commonly presents in lymph nodes, RDD can also present in extranodal sites including the respiratory tract, skin, bone, genitourinary tract, central nervous system, bone marrow, visceral organs, and soft tissues [1, 2]. Among extranodal RDD cases, only a minority present in the soft tissue [6]. While soft tissue RDD and nodal RDD exhibit some similarities, soft tissue lesions often have less distinct features, thereby complicating the identification and diagnosis of these lesions [5, 6]. This report aims to present a case of soft tissue RDD and review the current literature regarding the presentation, diagnostic findings, and treatment of this rare histiocytosis.

## 2. Statement of Informed Consent

The patient was informed that data concerning the case would be submitted for publication, and she provided consent.

## 3. Surgical Case Report Guidelines

This case report has been reported in line with the Surgical Case Report (SCARE) 2020 criteria [7].

## 4. Presentation of Case

**Figure 1:**
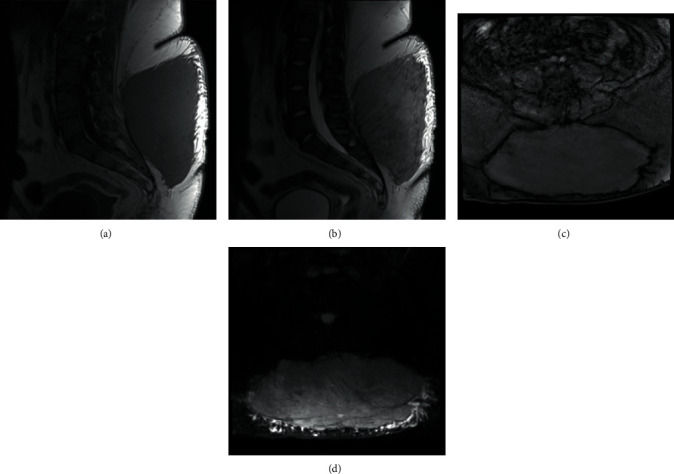
Representative preoperative 1.5 Tesla MRI images. (a) T1 TSE sagittal (TR 625 ms, TE 13 ms). (b) T2 TSE sagittal (TR 4160 ms, TE 97 ms). (c) T1 VIBE FS axial (TR 5.07, TE 2.39 ms). (d) T2 TSE FS axial (TR 5080 ms, TE 81 ms). These images revealed a large mass in the subcutaneous tissues of the lower back. The mass measured 24.8 cm × 10.4 cm × 20.5 cm in transverse, anteroposterior, and caudocranial dimensions. It extended in close proximity to the superficial fascial plane over the paraspinal musculature. No definite muscular involvement was identified. The lesion demonstrated high signal on the T2-weighted sequences and intermediate signal on the T1-weighted sequences. TSE = turbo spin echo, FS = fat suppression, VIBE = volumetric interpolated breath-hold examination, TR = repetition time, and TE = echo time.

An 18-year-old female presented to the orthopedic clinic with a chief complaint of a slow-growing mass located in her lower back for the past year. A firm, nontender large superficial soft tissue mass that was approximately 25 centimeters long and 12 centimeters wide overlying the lumbar spine was noted. The mass appeared to be mostly fixed to underlying structures. There were no skin or surface changes overlying her mass, and no lymphadenopathy was noted on physical examination. An MRI of the lumbar spine demonstrated a large, intermediate T1/T2-weighted lesion in the superficial subcutaneous tissue abutting the deep fascia of the lumbosacral spine (Figure 1). The patient subsequently underwent a CT guided-biopsy of the lumbosacral mass utilizing an Achieve co-axial biopsy device (Merit Medical, Jordan, UT, USA). Histologic examination of the core biopsy samples revealed spindled and histiocyte-like cells that expressed S100 protein and CD163 by immunohistochemistry. The spindled cells were also negative for smooth muscle actin, nuclear beta-catenin, CD34, and keratin AE1/3. Throughout the lesional tissue, p16 was negative and p53 showed a wild-type (normal) staining pattern. Cyclin D1 expression was focal and weak. MDM2 amplification was absent by fluorescence in situ hybridization (FISH). Additionally, there was a chronic perivascular inflammatory component consisting of a mixture of CD3-positive T cells and CD20-positive B cells. Given the histology, absence of p53 and p16 overexpression, lack of MDM2 amplification, and low Ki-67 proliferative index (<1%), a benign process was favored.

With the pathology of the soft tissue biopsy, the differential diagnosis included a regressing RDD as well as a localized massive lymphedema. Although emperipolesis was not seen on the pathology specimen and cyclin D1 was only focally expressed, the leading diagnosis remained RDD. With these results, treatment options (including operative and nonoperative intervention) were discussed at length with the patient, who ultimately wished to proceed with surgical excision of her lumbosacral mass.

The patient was brought into the operating room by the senior author (DML) and placed prone on the operating room table. A straight 20-centimeter midline incision was made over the mass. Dissection was carried to the lesion, which was noted to be fibrous in nature. The mass was subsequently meticulously shelled out with electrocautery and sent to pathology. The wound was closed in several layers—with a deep Jackson-Pratt (JP) drain left in the wound bed—with 0 Vicryl (Ethicon, Raritan, NJ, USA) for the deep fascial flaps, 2-0 Vicryl (Ethicon, Raritan, NJ, USA) for the subdermal layer, and a running 3-0 Monocryl (Ethicon, Raritan, NJ, USA) subcuticular closure. The wound was subsequently dressed with a PREVENA wound vac (3 M, St. Paul, MN, USA). A pressure bolster was placed on top and an abdominal binder used for reinforcement. The patient's postoperative course was uneventful, and she was not placed on any activity restrictions. The JP drain was removed on the second postoperative day, and the patient was subsequently discharged.

**Figure 2:**
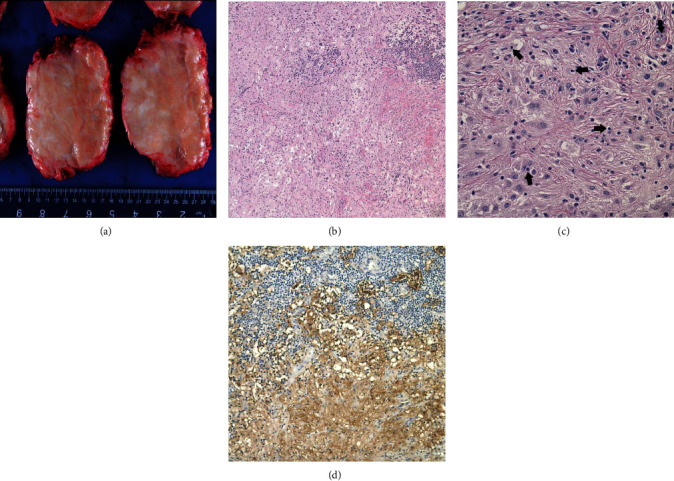
Pathologic analysis of the specimen. (a) Grossly, the lesion was well-circumscribed, unencapsulated, and tan-white in appearance. (b) Microscopically, the lesion was characterized by the presence of “light” (histiocyte-rich) and “dark” (lymphocyte-rich) areas. (Hematoxylin & eosin [H&E], 100×). (c) At higher magnification, emperipolesis (arrows) is appreciated, a classic finding in RDD. (H&E, 400×). (d) An S100 immunohistochemical stain highlights the histiocytes in both the light (bottom) and dark (top) areas, further supporting the diagnosis of RDD (S100 immunostain, 200×).

The resected mass was unencapsulated but well defined with a tan-white, firm cut surface (Figure 2(a)). A microscopic examination showed sheets of enlarged histiocytes with clear to lightly eosinophilic cytoplasm admixed with smaller histiocytes and a chronic lymphoplasmacytic infiltrate with clusters of plasma cells (Figure 2(b)). Emperipolesis of lymphocytes and plasma cells was identified within the large histiocytes, which were S100+ by immunohistochemistry (Figures 2(c) and 2(d)). A CD163 stain highlighted both the large and small histiocytes. The Grocott-Gomori's methenamine silver (GMS) and acid-fast stains did not reveal any organisms within the lesion. Given these findings, the final pathologic diagnosis for the patient's lumbosacral mass was soft tissue RDD.

**Figure 3:**
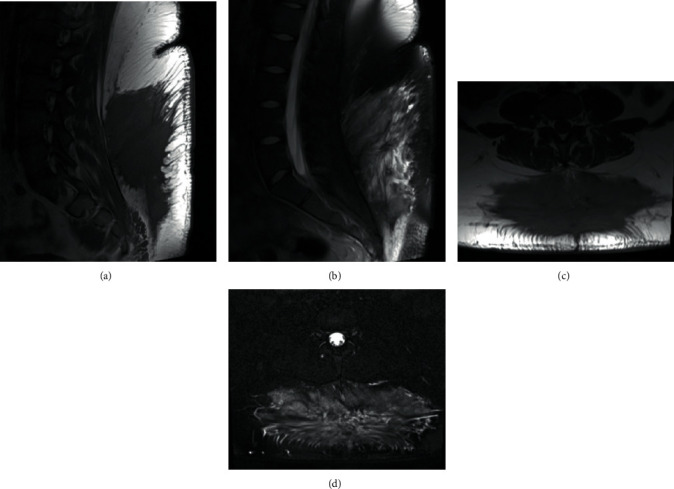
Representative 9-month postoperative 1.5 Tesla MRI images. (a) T1 sagittal (TR 500 ms, TE 17 ms). (b) T2 FS sagittal (TR 4,770 ms, TE 83 ms). (c) T1 axial (TR 600 ms, TE 19 ms). (d) T2 FS axial (TR 6,580 ms, TE 100 ms). These images revealed a large T1 hypointense, T2 heterogeneously hyperintense, and enhancing lesion with irregular borders, measuring approximately 21.5 × 7 × 15 cm (transverse × anteroposterior × craniocaudal). There was a central stellate/spiculated portion of the lesion, which was a relatively nonenhancing region and suggestive of an area of postsurgical scarring. Surrounding this region was a more T2 hyperintense multilobulated appearing component. No other lesions were seen. FS = fat suppression, TR = repetition time, and TE = echo time.

At the patient's two-week postoperative visit, she was doing well without any complaints. The patient had developed a large seroma at the operative site, but the incision had healed well. At the patient's six-week postoperative visit, she reported less pain and increased motion of her lumbosacral spine. The seroma was still present, but it remained soft and nontender. Nine months following her operation, an MRI of the lumbar spine (Figure 3) demonstrated a large T1 hypointense, T2 heterogeneously hyperintense, and enhancing lesion with irregular borders and central area of scarring. The patient remained asymptomatic at this point in time, and a decision was made to continue to monitor the patient and her symptoms. Seventeen months following the patient's operation, the patient denied any pain or limitations in the range of motion of her lumbosacral spine.

## 5. Discussion

Although nodal RDD is most common form of the disease, 43% of RDD cases involve at least one extranodal site, with 23% of cases presenting exclusively in extranodal sites [3]. These sites include the respiratory tract, skin, bone, genitourinary tract, central nervous system, bone marrow, visceral organs, and soft tissues [1, 2]. Among extranodal RDD cases, only a minority of cases present in the soft tissue, as evidenced by the fact that a total of 36 cases of soft tissue RDD were reported from 1969 to 2013 [6]. Clinically, patients with soft-tissue RDD most oftenly present with an asymptomatic mass in soft tissue regions, including the extremities, trunk, head, and neck [3]. However, because soft tissue RDD often presents with less distinct features than nodal RDD, identification and diagnosis of lesions can be more complicated [5, 6].

The diagnosis of RDD was determined based on the radiological, gross, histological, and immunohistochemical findings. Histologically, RDD is generally characterized by emperipolesis and a mixed infiltrate of S100+ histiocytes, mononuclear cells, plasma cells, and lymphocytes. These histiocytes, also called “RDD cells,” contain abundant clear to pale eosinophilic cytoplasm and small, central nuclei with eosinophilic nucleoli [1]. Emperipolesis is the presence of intact lymphocytes within the histiocyte cytoplasm [1–6, 8, 9]. Histiocytes may also contain engulfed plasma cells, neutrophils, and red blood cells. Extranodal RDD may demonstrate characteristic alternating pale (histiocyte-rich) and dark (lymphocyte-rich) regions with sheets of eosinophilic, polygonal to spindled histiocytes arranged in a storiform pattern [2, 3, 5]. Although soft tissue RDD may histologically resemble nodal RDD, soft tissue RDD also demonstrates some notable histologic differences [1]. These include the lack of nodal architecture, the presence of increased fibrosis and collagen deposition, and generally fewer RDD cells [1]. The presence of emperipolesis is also typically less distinct due to poorly defined cell boundaries [2, 3, 5]. Immunohistochemically, RDD histiocytes are strongly S100+ and CD163+, variably CD68+, and negative for CD1a and HLA-DR [1, 3–6, 8]. Of these various gross, histological, and immunohistochemical features, the hallmark diagnostic criteria are the presence of S100+ RDD histiocytes and emperipolesis [1–9]. For soft tissue lesions, which often have less specific findings, polygonal RDD histiocytes are especially important [3].

The differential diagnosis for RDD includes Langerhans cell histiocytosis (LCH), soft tissue fibrohistiocytic lesions, histiocytic sarcomas, and myxoinflammatory fibroblastic sarcomas. LCH may occur in similar sites as RDD, but these lesions exhibit Langerhans cells with grooved nuclei, eosinophils, and Birbeck granules [10]. Additionally, although LCH also consists of S100+ cells, it is the Langerhans cells—not the histiocytes—that are S100+ in LCH. Langerhans cells also stain strongly for CD1a and do not demonstrate emperipolesis [11]. Histiocytes found in fibrohistiocytic lesions of soft tissue—including benign fibrous histiocytoma and dermatofibrosarcoma protuberans—demonstrate higher nuclear to cytoplasmic ratios, more hyperchromatic nuclei, and a more whorled pattern than RDD lesions [3, 6]. Histiocytic sarcomas exhibit malignant features that are not found in RDD, including marked cytologic atypia and increased mitotic activity. Additionally, histiocytic sarcoma cells are often HLA-DR positive and CD30 negative [9, 11]. Although histiocytic sarcoma may rarely present with hemophagocytosis, these engulfed cells are no longer viable. In contrast, emperipolesis found in RDD is characterized by the presence of viable cells within histiocytic cytoplasm [12]. Myxoinflammatory fibroblastic sarcoma may also exhibit histiocyte-like cells within an inflammatory background, but these lesions consist of myxoid areas with numerous pseudolipoblasts and nuclear atypia and are S100 negative [9, 11]. Sarcoidosis does not exhibit emperipolesis or S-100 staining and often presents with pulmonary involvement [11]. Due to these differentiating characteristics, the findings of the case led to a diagnosis of STRDD.

Although a treatment strategy for patients with extranodal RDD has been described [13], at this time, there is no uniform approach, and patients are treated based on individual clinical circumstances [13]. Extranodal RDD most often resolves spontaneously without intervention, but can cause morbidity and death via involvement to vital organs. If spontaneous remission does not occur, corticosteroids, chemotherapy, radiotherapy, and alkaloids may be utilized, but the efficacy of these treatments remains inconclusive [2, 3]. Surgical debulking and removal has been demonstrated to be the best option to prevent recurrence of the mass [1, 4, 6], as was the treatment strategy in this case. Although the patient developed a large seroma and also demonstrated a large lesion in her nine-month follow-up MRI, the patient denied any pain or loss of range of motion and returned to all activities without limitations. The patient will continue to be followed clinically as well as radiographically.

## 6. Conclusion

RDD is a rare, benign type II histiocytosis characterized by emperipolesis—the presence of intact lymphocytes within the histiocyte cytoplasm—and a mixed infiltrate of S100+ histiocytes, mononuclear cells, plasma cells, and lymphocytes. Although soft tissue RDD may histologically resemble nodal RDD, soft tissue RDD also demonstrates some notable histologic differences including the lack of nodal architecture, the presence of increased fibrosis and collagen deposition, and generally fewer RDD cells. This case presentation demonstrates one few reports of isolated soft tissue RDD within the lumbosacral region without associated lymphadenopathy or skin changes and highlights the heterogeneity that still exists in the treatment paradigm of extranodal RDD.

## Data Availability

No datasets were generated or analyzed during the current study.

## Additional Points

*Highlights.* (i) RDD is a rare, benign type II histiocytosis characterized by emperipolesis—the presence of intact lymphocytes within the histiocyte cytoplasm—and a mixed infiltrate of S100+ histiocytes, mononuclear cells, plasma cells, and lymphocytes. (ii) Although soft tissue RDD may histologically resemble nodal RDD, soft tissue RDD also demonstrates some notable histologic differences including the lack of nodal architecture, the presence of increased fibrosis and collagen deposition, and generally fewer RDD cells. (iii) At this time, there is no standardized treatment strategy for the management of patients with extranodal RDD, and patients are treated based on individual clinical circumstances. *Research Registration.* This is not a “first in man” study, and therefore this work has not been submitted with a Research Registry Unique Identifying Number. *Guarantor.* The guarantor of the publication is Alana M Munger, MD.
